# The cases of rapid progression of kypho-scoliosis in adults

**DOI:** 10.1186/1748-7161-10-S1-P2

**Published:** 2015-01-19

**Authors:** Koji Kanzaki, Keizo Sugisaki, Junichi Ochiai, Takayuki Nakajima, Kazuyuki Segami

**Affiliations:** 1 Department of Orthopaedic Surgery, Showa University Fujigaoka Hospital, Japan

## Purpose

In most patients degenerative kypho-scoliosis progresses slowly, because the progress of deformity depends on the degeneration of the spine associated with aging. In some cases, a rapid progression of kypho-scoliosis was observed. Eight cases with this rapid progression of kypho-scoliosis in patients over 60 years of age were investigated clinically and radiographically in this study. The purpose of this study was to clarify the differences of the etiology of rapid progression of deformity in these cases.

## Materials and method

Materials used in this study were 8 cases (2 male and 6 female). Kyphosis was observed in 3 cases, and kypho-scoliosis in 5 cases. Cobb angle, C7 offset, LL, PI, PT, SS, and SVA were measured in whole spine X-rays, as well as conditions of PVM were investigated in MRIs. Three cases were treated surgically.

## Results

The average angle of LL, PT, and SS was -4.5, 36.5, and 16.5 degrees, the average SVA was 192mm, the average Cobb angle was 39.4 degrees, and average C7 offset was 140mm. Back pain preceding the progression of deformity was observed in all cases. Parkinson's disease was observed previously in one case, and in a different case was reported during follow-ups. The mottled T2 high views were observed in all cases except in both of these Parkinson's disease cases. The uni-lateral T2 high views in coronal off-set sides were observed in all kypho-scoliosis cases. Three cases were treated surgically with instrumentation in order to correct and fuse. As a result, good clinical results were obtained in these cases.

## Discussion

The progression of deformity with degenerative kypho-scoliosis in adults is generally slow. In cases of rapid progression of deformity in adults, every case obtained significant SVA or C7 offset with unfavorable global balance in this study. Something is occurring in the tissues around the degenerative spine in these cases, because the progression of deformity depends on the degeneration of the spine associated with the progress of aging. The MRI findings in this study suggest that the dysfunction of PVM could be leading to the rapid progression of this deformity. The load of the PVM increases gradually with the degeneration of the spine, and the breakdown of the spine can occur as a result. Because of this, the progression of the deformity of the spine will no longer be controlled by those PVM.

## Conclusion

Eight cases of rapid progression in adult kypho-scoliosis were reported.

